# Charting immune variation through genetics and single-cell genomics

**DOI:** 10.1093/gigascience/giaf161

**Published:** 2026-01-08

**Authors:** Joseph E Powell

**Affiliations:** Translational Genomics, Garvan Institute of Medical Research, Translational Genomics, Darlinghurst, Sydney, NSW 2021, Australia; UNSW Cellular Genomics Futures Institute, University of New South Wales, Kensington, Sydney 2052, Australia

## Abstract

Large-scale single-cell genomics projects have revolutionized our understanding of human immune variation. Yet most studies to date have been Eurocentric, limited in cell-type resolution, or restricted to a single data modality. The newly published Chinese Immune Multi-Omics Atlas helps address these gaps by profiling 428 healthy Chinese adults using a multiomics single-cell approach that combines single-cell RNA sequencing and single-cell chromatin accessibility sequencing across over 10 million immune cells. This integrated strategy enabled the identification of 73 distinct immune cell subsets and the construction of cell-type–specific gene regulatory networks linking noncoding enhancers to target genes. The atlas delineated hundreds of enhancer modules (eRegulons), highlighting both established and novel regulators of immune cell identity. By aligning transcriptomic and epigenomic maps, Yin et al. show how expanding both the ancestral diversity and data modalities of immune cell genomics can reveal new biology and provide a valuable addition to global reference cell atlases.

## Capturing Genetic and Geographic Diversity

Reporting in *Science*, Yin et al. [1] present the Chinese Immune Multi-Omics Atlas (CIMA), a study that focuses on an East Asian (Chinese) cohort, bringing much-needed diversity to population genetic single-cell research and cell atlases. This data resource complements other recent Asian immune cohort projects, such as the multinational Asian Immune Diversity Atlas, which has profiled single-cell RNA from circulating immune cells across diverse Asian populations [2], and the ImmuNexUT program, which generated bulk RNA data from Fluorescence-Activated Cell Sorting (FACS)-sorted immune cells in a Japanese cohort [3]. Against this backdrop, CIMA stands out as one of the first large-scale immune multiomics resources centered on an East Asian population, offering integrative genomic and epigenomic data at a considerable scale.

CIMA’s multiomics analyses reveal apparent population-specific genetic effects. For instance, over 93% of CIMA’s *cis*-expression Quantitative Trait Loci (eQTL) target genes overlapped with the Japanese ImmuNexUT immune eQTL database [3], whereas only ~44% overlapped with the European-derived OneK1K dataset [4]. This disparity highlights how genetic influences on immune gene expression can differ markedly between ancestries. Indeed, many regulatory variants common in Asian populations are rare in Europeans and vice versa.

One example is rs11886530 (chr2:100,809,622), which is common in East Asians (minor allele frequency ~0.38) but rare in Europeans (~0.06). In CIMA’s cohort, this East Asian–enriched allele drives a *cis*-effect on the *NPAS2* gene in T cells and a concurrent *trans*-effect on *NR1D1*—2 core circadian clock genes whose coordinated regulation had not been identified in immune cells before this study. By broadening genetic ancestral diversity, CIMA and similar efforts are uncovering biological connections that would likely be missed in predominantly European cohorts.

## Resolving Immune Cell States and Genetic Effects

A significant challenge in immunogenomics has been the resolution of cell types and states from which genomic information is generated. Traditional (bulk) studies rely on cell sorting based on a limited number of canonical marker proteins [5], whereas single-cell resolution primarily depends on the number of cells per donor. Yin et al. [1] address this by mapping genetic influences at single-cell resolution across 73 finely resolved immune subsets, achieved by generating single-cell RNA sequencing (scRNA-seq) data on an average of 15,478 cells per donor and 9,602 cells per donor for scATAC. Noting that the discovery power for single-cell genetic effects drops for rarer cell types [6], the study identifies thousands of *cis*-eQTLs and over 50,000 *cis*-chromatin accessibility Quantitative Trait Loci (caQTLs) by analyzing each cell type separately. Strikingly, nearly 29% of the eGenes and 55% of the caQTLs were exclusive to a single cell type, underscoring that many regulatory variants are silent in most cells yet active in particular cell subsets. Even QTLs shared across multiple cell types often showed different effect sizes, reflecting subtle context dependencies. Together, these observations reinforce the evidence that genomic signals that are often obscured in bulk samples become clear when examined at the appropriate cellular resolution.

Yin et al. [1] illustrated this principle by pinpointing the cellular source of disease-linked genetic signals. A notable example is a sc-eQTL for *PADI2—*a gene implicated in rheumatoid arthritis—that was detected in whole blood but whose origin was ambiguous in previous studies [7]. CIMA revealed that this variant’s effect on *PADI2* expression derives almost entirely from *CD14* monocytes rather than from all leukocytes, despite being expressed ubiquitously across the myeloid lineage. Without single-cell resolution, that monocyte-specific signal was diluted in bulk blood. Pinpointing it to monocytes refines our understanding of *PADI2*’s role in autoimmunity, indicating that its contribution to rheumatoid arthritis is mediated through monocyte pathways rather than lymphocyte pathways.

## Linking Regulatory Variants to Disease Traits

CIMA further integrated its sc-e/caQTL results with genome-wide association studies (GWASs) to link genetic variants to clinical phenotypes [8]. Through this analysis, they were able to identify ~1,200 significant variant→gene or variant→chromatin→trait associations across more than 150 immune-related traits. Aligning with the increasing evidence of cell specificity of genetic effects, ~73% of these associations were attributable to a single immune cell type, highlighting that for most immune traits, the causal variant’s impact is channeled through one specific cellular niche. For example, a GWAS variant associated with asthma and autoimmunity influences circulating IL-12B levels by altering *IKZF4* expression specifically in CD4 Treg-FOXP3 cells. *IKZF4* is critical for Treg function, so this Treg-specific effect links a DNA polymorphism to dysregulated IL-12/IL-23 signaling implicated in asthma and autoimmunity. These findings demonstrate that many immune disease risk alleles exert their effects through cell-type–specific regulatory mechanisms, consistent with the observation that numerous disease loci map to enhancers active only in particular immune cells and with previous results from scRNA-seq cohorts [2, 4].

## Conclusion

### The value of intersecting human genetics and cellular genomics

The convergence of large-scale cell atlases and population genomics is enabling new insights into how natural genetic variation shapes human immune cell states. Integrating genetic association data with high-resolution single-cell phenotyping allows researchers to move beyond descriptive atlases toward predictive, mechanistic models of immune function [6]. The work of Yin et al. [1] provides further evidence of this shift: its deep profiling of genetically diverse individuals, combined with regulatory mapping across gene expression and chromatin accessibility, creates a valuable resource for linking inherited variation to molecular phenotypes.

Beyond immediate applications in variant interpretation and disease mapping, such datasets will increasingly inform the next generation of computational frameworks, including large foundation models of cell states. Current cell models are typically trained on perturbation-based datasets that often rely on artificial overexpression or CRISPR interference to infer regulatory networks. However, natural genetic variation perturbs molecular pathways more subtly and across many individuals, offering a complementary and physiologically relevant source of signal. Incorporating large-scale population variation into training data will enhance the generalizability and biological fidelity of foundation models, especially when these models are used to simulate disease processes or predict therapeutic responses.

In this context, multiomic resources such as CIMA do more than catalog immune cell states; they also help define the functional axes along which these states vary across individuals. As the field moves toward building generative models of human biology, the intersection of genetic diversity and cellular resolution will be essential not only for representation but also for inference.

## Supplementary Material

giaf161_Supplemental_File

## Data Availability

Not applicable.

## Abbreviations

CIMA: Chinese Immune Multi-Omics Atlas; GWAS: genome-wide association studies; scRNA-seq: single-cell RNA sequencing.
