# Supplementary material for: Charting immune variation through genetics and single-cell genomics
Source: Gigascience. 2026 Jan 8;15:giaf161. doi: 10.1093/gigascience/giaf161 (PMC12821369; doi:10.1093/gigascience/giaf161)
Supplement: giaf161_Supplemental_File [file giaf161_supplemental_file.pdf]

GigaScience

Charting Immune Variation Through Genetics and Single-Cell Genomics

--Manuscript Draft--

**Manuscript Number:**	GIGA-D-25-00509
**Full Title:**	Charting Immune Variation Through Genetics and Single-Cell Genomics
**Article Type:**	Commentary
**Funding Information:**	
**Abstract:**	Large-scale single-cell genomics projects have revolutionised our understanding of human immune variation. Yet most studies to date have been Eurocentric, limited in cell-type resolution, or restricted to a single data modality1-4. The newly published Chinese Immune Multi-Omics Atlas (CIMA) helps address these gaps by profiling 428 healthy Chinese adults using a multi-omics single-cell approach that combines single-cell RNA sequencing (scRNA-seq) and single-cell chromatin accessibility sequencing (scATAC-seq) across over 10 million immune cells5. This integrated strategy enabled the identification of 73 distinct immune cell subsets and the construction of cell-type–specific gene regulatory networks linking noncoding enhancers to target genes. The atlas delineated hundreds of enhancer modules (eRegulons), highlighting both established and novel regulators of immune cell identity. By aligning transcriptomic and epigenomic maps, Yin et al. show how expanding both the ancestral diversity and data modalities of immune cell genomics can reveal new biology and provide a valuable addition to global reference cell atlases5.
**Corresponding Author:**	Joseph Powell<br><br>AUSTRALIA
**Corresponding Author Secondary Information:**	
**Corresponding Author's Institution:**	
**Corresponding Author's Secondary Institution:**	
**First Author:**	Joseph Powell
**First Author Secondary Information:**	
**Order of Authors:**	Joseph Powell
**Order of Authors Secondary Information:**	
**Additional Information:**	
**Question**	**Response**
Are you submitting this manuscript to a special series or article collection?	No
**Experimental design and statistics**<br><br>Full details of the experimental design and statistical methods used should be given in the Methods section, as detailed in our Minimum Standards Reporting Checklist. Information essential to interpreting the data presented should be made available in the figure legends.	No

Powered by Editorial Manager® and ProduXion Manager® from Aries Systems Corporation

Have you included all the information requested in your manuscript?	
If not, please give reasons for any omissions below.<br><br>as follow-up to "**Experimental design and statistics**<br><br>Full details of the experimental design and statistical methods used should be given in the Methods section, as detailed in our Minimum Standards Reporting Checklist. Information essential to interpreting the data presented should be made available in the figure legends.<br><br>Have you included all the information requested in your manuscript?<br><br>"	It is a commentary
**Resources**<br><br>A description of all resources used, including antibodies, cell lines, animals and software tools, with enough information to allow them to be uniquely identified, should be included in the Methods section. Authors are strongly encouraged to cite Research Resource Identifiers (RRIDs) for antibodies, model organisms and tools, where possible.<br><br>Have you included the information requested as detailed in our Minimum Standards Reporting Checklist?	No
If not, please give reasons for any omissions below.<br><br>as follow-up to "**Resources**<br><br>A description of all resources used, including antibodies, cell lines, animals	It is a commentary

and software tools, with enough information to allow them to be uniquely identified, should be included in the Methods section. Authors are strongly encouraged to cite Research Resource Identifiers (RRIDs) for antibodies, model organisms and tools, where possible.<br><br>Have you included the information requested as detailed in our Minimum Standards Reporting Checklist?<br><br>"	
**Availability of data and materials**<br><br>All datasets and code on which the conclusions of the paper rely must be either included in your submission or deposited in publicly available repositories (where available and ethically appropriate), referencing such data using a unique identifier in the references and in the "Availability of Data and Materials" section of your manuscript.<br><br>Have you have met the above requirement as detailed in our Minimum Standards Reporting Checklist?	No
If not, please give reasons for any omissions below.<br><br>as follow-up to "**Availability of data and materials**<br><br>All datasets and code on which the conclusions of the paper rely must be either included in your submission or deposited in publicly available repositories (where available and ethically appropriate), referencing such data using a unique identifier in the references and in the "Availability of Data and Materials" section of your manuscript.	It is a commentary.

Powered by Editorial Manager® and ProduXion Manager® from Aries Systems Corporation

Have you have met the above requirement as detailed in our Minimum Standards Reporting Checklist?<br><br>"	
GigaScience has policies and guidelines in place for the use of generative AI-writing tools such as ChatGPT. If you have used such writing tools to assist with writing the manuscript this must be declared and cited in the text. Authors should not list AI-writing tools and other AI-assisted technologies as an author or co-author and should acknowledge that they are fully responsible for text generated or refined by AI-writing tools.<p><br><br>A summary of use (particularly in the introduction or among methods) needs to be included at the end of the paper, and the outputs should also be included as a supplementary file hosted in GigaDB or other open repositories. Please read our guidelines for more information. <p><br><br>By submitting to GigaScience, you are aware of the journal's AI-writing tools policy, and if you have declared use of such tools below, you have acknowledged this where appropriate in your manuscript and have made a summary of use and outputs available. <p><br><b>AI-assisted writing tools have been used in the preparation of this manuscript?	No

Manuscript
Click here to access/download;Manuscript;Powell_commentary_CIMA_Scie

Charting Immune Variation Through Genetics and Single-Cell Genomics

Joseph E. Powell[1,2]

1. Translational Genomics, Garvan Institute of Medical Research, Darlinghurst, Sydney, NSW, 2021, Australia
2. UNSW Cellular Genomics Futures Institute, University of New South Wales, Kensington, Sydney, 2052, Australia

Corresponding: j.powell@garvan.org.au

Abstract

Large-scale single-cell genomics projects have revolutionised our understanding of human immune variation. Yet most studies to date have been Eurocentric, limited in cell-type resolution, or restricted to a single data modality. The newly published Chinese Immune Multi-Omics Atlas (CIMA) helps address these gaps by profiling 428 healthy Chinese adults using a multi-omics single-cell approach that combines single-cell RNA sequencing (scRNA-seq) and single-cell chromatin accessibility sequencing (scATAC-seq) across over 10 million immune cells. This integrated strategy enabled the identification of 73 distinct immune cell subsets and the construction of cell-type–specific gene regulatory networks linking noncoding enhancers to target genes. The atlas delineated hundreds of enhancer modules (eRegulons), highlighting both established and novel regulators of immune cell identity. By aligning transcriptomic and epigenomic maps, Yin *et al*. show how expanding both the ancestral diversity and data modalities of immune cell genomics can reveal new biology and provide a valuable addition to global reference cell atlases.

Capturing Genetic and Geographic Diversity

CIMA's focus on an East Asian (Chinese) cohort brings much-needed diversity to population genetic single-cell research and cell atlases. The data resource complements other recent Asian immune cohort projects, such as the multinational Asian Immune Diversity Atlas (AIDA), which has profiled single-cell RNA from circulating immune cells across diverse Asian populations[1], and the ImmuNexUT program, which generated bulk RNA data from FACS-sorted immune cells in a Japanese cohort[2]. Against this backdrop, CIMA stands out as one of the first large-scale immune multi-omics resources centred on an East Asian population, offering integrative genomic and epigenomic data at a considerable scale.

CIMA's multi-omics analyses reveal apparent population-specific genetic effects. For instance, over 93% of CIMA's *cis*-eQTL target genes overlapped with the Japanese ImmuNexUT immune eQTL database[2], whereas only ~44% overlapped with the European-derived OneK1K dataset[3]. This disparity highlights how genetic influences on immune gene expression can differ markedly between ancestries. Indeed, many regulatory variants common in Asian populations are rare in Europeans, and vice versa.

One example is rs11886530 (chr2:100809622) that is common in East Asians (minor allele frequency ~0.38) but rare in Europeans (~0.06). In CIMA's cohort, this East Asian–enriched allele drives a *cis*-effect on the *NPAS2* gene in T cells and a concurrent *trans*-effect on *NR1D1* – two core circadian clock genes whose coordinated regulation had not been identified in immune cells before this study. By broadening genetic ancestral diversity, CIMA and similar efforts are uncovering biological connections that would likely be missed in predominantly European cohorts.

Resolving Immune Cell States and Genetic Effects

A significant challenge in immunogenomics has been the resolution of cell types and states from which genomic information is generated. Traditional (bulk) studies rely on cell sorting based on a limited number of canonical marker proteins[4], whereas single-cell resolution primarily depends on the number of cells per donor. Yin *et al.* address this by mapping genetic influences at single-cell resolution across 73 finely resolved immune subsets, achieved by generating scRNA-seq data on an average of 15,478 cells per donor and 9,602 cells per donor for scATAC. Noting that the discovery power for single-cell genetic effects drops for rarer cell types[5], the study identifies thousands of *cis*-eQTLs and over 50,000 *cis*-caQTLs by analysing each cell type separately. Strikingly, nearly 29% of the eGenes and 55% of the caQTLs were exclusive to a single cell type, underscoring that many regulatory variants are silent in most cells yet active in particular cell subsets. Even QTLs shared across multiple cell types often showed different effect sizes, reflecting subtle context dependencies. Together, these observations reinforce the evidence that genomic signals that are often obscured in bulk samples become clear when examined at the appropriate cellular resolution.

Yin *et al.* illustrated this principle by pinpointing the cellular source of disease-linked genetic signals. A notable example is a sc-eQTL for *PADI2* – a gene implicated in rheumatoid arthritis – that was detected in whole blood but whose origin was ambiguous in previous studies[6]. CIMA revealed that this variant's effect on *PADI2* expression derives almost entirely from *CD14* monocytes rather than from all leukocytes, despite being expressed ubiquitously across the myeloid lineage. Without single-cell resolution, that monocyte-specific signal was diluted in bulk blood. Pinpointing it to monocytes refines our understanding of *PADI2*'s role in autoimmunity, indicating that its contribution to rheumatoid arthritis is mediated through monocyte pathways rather than lymphocyte pathways.

Linking Regulatory Variants to Disease Traits

CIMA further integrated its sc-e/caQTL results with genome-wide association studies (GWAS) to link genetic variants to clinical phenotypes[7]. Through this analysis, they were able to identify ~1,200 significant variant→gene or variant→chromatin→trait associations across more than 150 immune-related traits. Aligning with the increasing evidence of cell specificity of genetic effects, ~73% of these associations were attributable to a single immune cell type, highlighting that for most immune traits, the causal variant's impact is channelled through one specific cellular niche. For example, a GWAS variant associated with asthma and autoimmunity influences circulating IL-12B levels by altering *IKZF4* expression specifically in CD4 Treg-FOXP3 cells. *IKZF4* is critical for Treg function, so this Treg-specific effect links a DNA polymorphism to dysregulated IL-12/IL-23 signalling implicated in asthma and autoimmunity. These findings demonstrate that many immune disease risk alleles exert their effects through cell-type–specific regulatory mechanisms, consistent with the observation that numerous disease loci map to enhancers active only in particular immune cells and with previous results from scRNA-seq cohorts[1,3].

Conclusion

The value of intersecting human genetics and cellular genomics

The convergence of large-scale cell atlases and population genomics is enabling new insights into how natural genetic variation shapes human immune cell states. Integrating genetic association data with high-resolution single-cell phenotyping allows researchers to move beyond descriptive atlases toward predictive, mechanistic models of immune function[5]. The work of Yin et al. provides further evidence of this shift: its deep profiling of genetically diverse individuals, combined with regulatory mapping across gene expression and chromatin accessibility, creates a valuable resource for linking inherited variation to molecular phenotypes.

Beyond immediate applications in variant interpretation and disease mapping, such datasets will increasingly inform the next generation of computational frameworks, including large foundation models of cell states. Current cell models are typically trained on perturbation-based datasets that often rely on artificial overexpression or CRISPR interference to infer regulatory networks. However, natural genetic variation perturbs molecular pathways more subtly and across many individuals, offering a complementary and physiologically relevant source of signal. Incorporating large-scale population variation into training data will enhance the generalizability and biological fidelity of foundation models, especially when these models are used to simulate disease processes or predict therapeutic responses.

In this context, multi-omic resources such as CIMA do more than catalogue immune cell states; they also help define the functional axes along which these states vary across individuals. As the field moves toward building generative models of human biology, the intersection of genetic diversity and cellular resolution will be essential not only for representation but for inference.

Data Availability

Not applicable

Competing Interests

None

References

1. Kock K., et al. Asian diversity in human immune cells. Cell, 188, (2025). DOI: 10.1016/j.cell.2025.02.017

2. Ota M., et al. Dynamic landscape of immune cell-specific gene regulation in immune-mediated diseases. Cell, 184, (2021). DOI: 10.1016/j.cell.2021.03.056

3. Yazar S., et al. Single-cell eQTL mapping identifies cell type-specific genetic control of autoimmune disease. Science, 376, (2022). DOI: 10.1126/science.abf3041

4. Schmiedel B., et al. Impact of genetic polymorphisms on human immune cell gene expression. Cell, 175, (2018). DOI: 10.1016/j.cell.2018.10.022

5. Cuomo A., et al. Single-cell genomics meets human genetics. Nature Reviews Genetics, 24, (2023). DOI: 10.1038/s41576-023-00599-5

6. Goldman K., et al. Expression quantitative trait loci analysis in rheumatoid arthritis identifies tissue specific variants associated with severity and outcome. Annals of the Rheumatic Diseases, 83, (2024). DOI: 10.1136/ard-2023-224540

7. Zhu Z., et al. Integration of summary data from GWAS and eQTL studies predicts complex trait gene targets. Nature Genetics, 48, (2016). DOI: 10.1038/ng.3538
